# Effect of Supplementation with the Combination of Se-Enriched *Lentinula edodes* Mycelium, Exogenous Enzymes, Acidifiers, Sodium Butyrate and Silicon Dioxide Nanoparticle Feed Additives on Selected Parameters in Calves

**DOI:** 10.3390/molecules27165163

**Published:** 2022-08-13

**Authors:** Ewelina Szacawa, Katarzyna Dudek, Magdalena Wasiak, Dariusz Bednarek, Dorota Bederska-Łojewska, Bożena Muszyńska, Marek Pieszka

**Affiliations:** 1 Department of Cattle and Sheep Diseases, National Veterinary Research Institute, 24-100 Pulawy, Poland; 2 Department of Pathology, National Veterinary Research Institute, 24-100 Pulawy, Poland; 3 Department of Animal Nutrition and Feed Science, National Research Institute of Animal Production, 32-083 Balice, Poland; 4 Department of Neurobiology, Institute of Pharmacology, Polish Academy of Sciences, 31-343 Krakow, Poland; 5 Department of Pharmaceutical Botany, Faculty of Pharmacy, Jagiellonian University Medical College, 30-688 Krakow, Poland

**Keywords:** feed additive, mycelial culture, *Lentinula edodes*, calves, selenium, immune response, cytokines, average daily gains

## Abstract

During the initial months of calves’ lives, the young animals are exposed to bacterial and viral infections, and during this period, crucial physiological changes take place in their organisms. Offering calves feed additives that will have a beneficial influence on their organisms and improve their growth while reducing the morbidity rate is the optimal task of feeding. This is the first study to investigate the effect of experimental supplementation for calves with the combination of two feed additives—one containing *Lentinula* *edodes* enriched with selenium (Se), and the second containing pancreatic-like enzymes, fat-coated organic acids, sodium butyrate, and silicon dioxide nanoparticles—on the serum Se concentration, selected immune parameters, and the average daily gains in the calves. During the study, the serum Se concentration was examined by means of inductively coupled plasma mass spectrometry, and the immunoglobulin and cytokine concentrations with ELISA assays. The white blood cell (WBC) count with leukocyte differentiation was examined with the use of a hematological analyzer, and the percentages of subpopulations of T lymphocytes and monocytes, phagocytic activity, and oxidative burst of monocytes and granulocytes with the use of a flow cytometer. The average daily gains of the calves were also evaluated. In summary, the supplementation of the experimental calves with the combination of two feed additives resulted in significantly higher serum Se concentrations, and the immune systems of the calves were not suppressed while the examined feed additives were being delivered. Although not statistically significant, some positive effects on the calves were seen: a tendency towards the improvement of some of the immune parameters evaluated, and a tendency for higher average daily gains in the calves.

## 1. Introduction

Calves lose their natural colostral immunity after weaning and start to develop their own immune response, and in this period, cattle husbandry encounters problems, such as infections caused by pathogenic microorganisms. The main source of both innate and adaptive immunity is white blood cells (WBC), and each type of these cells—lymphocytes (LYM), monocytes (MON), and granulocytes (GRA)—exhibits a specific function. WBC, with the function of antigen-presenting cells, present protein antigens in conjunction with class II major histocompatibility complex (MHC II) molecules, while T cells attack cells infected with bacteria or viruses. Other studies indicate a significant role for γδ T cells in the host defense against pathogens [1]. Their amount can be influenced by different physiological and pathological factors. Generally, a deficiency of the leukocyte (LEU) subpopulation and its dysfunction may result in increased susceptibility of cattle to different infections [2].

The occurrence of diseases in cattle often leads to a decrease in production or a reduced growth rate and performance. It is necessary to search for new additives that will have the optimal influence on calves’ organisms, reduce the morbidity rate, and improve their growth.

Many countries have banned the addition of antibiotics as growth promoters due to the increasingly widespread problem of bacterial drug resistance. However, investigations of novel feed additives that do not contain antibiotics have been conducted for a long time [3,4]. Such preparations should primarily improve calves’ digestion and support their optimal growth and performance. Offering calves feed additives that contain a mixture of compounds that have a bactericidal effect and will improve the digestion of complex nutrients will be reflected in the future good condition and long-term productivity of the animals [5]. On the other hand, proper immunological status is also important in cattle breeding.

Another problem that the cattle industry faces is a deficiency of selenium (Se) in the soil in some European countries and, as a result, Se deficiencies in feed produced in this soil. An examination shows that, although Se is added to complete feed, the serum selenium concentration in the animals has decreased in recent years in Europe [6,7]. It is a crucial trace element in feeding that is essential for the life and proper function of human and animal organisms. This mineral can influence the immune system and exhibit anti-inflammatory properties. All deviations from the normal range of Se concentration in the serum can lead to disease [8]. Looking specifically at cattle, deficiencies of this bioelement will contribute to mastitis, metritis, fertility problems and reduced milk yield in heifers, and muscular dystrophy in calves, which lead to economic losses [9,10,11]. The narrow therapeutic window and level of bioavailability of Se remain a challenge for scientists. Supplementation of this mineral with the organic form—selenitetriglycerides and selenomethionine—is more efficient than with the inorganic form [12,13,14,15]. Se is naturally found in animal and plant sources. Taking into consideration animal feeding, yeast and fruiting bodies of edible mushrooms are suitable organisms to use as supplements because they show a high capacity to accumulate microelements such as Se and, under some conditions, may have anti-inflammatory, antioxidative, and prebiotic properties [16,17,18,19,20]. It has been shown that β-glucan is one of the main functional compounds present in the mycelium of *Lentinula edodes* [21], and such a bioactive metabolite can, in some conditions, act as a health-promoting, immunomodulatory, and antioxidant substance. It can also influence the production of pro- and anti-inflammatory cytokines [22,23]. The safety of using *L. edodes* mycelium further enriched with organic Se was proven by a study by Muszyńska et al. [24], which concerned its production and the examination of the safety of its use. It revealed that these edible mushrooms, enriched with Se in the form of selenitetriglycerides, had no cytotoxic effect and can be used in cattle feeding to prevent Se deficiencies. Another study investigated the impact of a feed additive containing exogenous enzymes, acidifiers, sodium butyrate, and nSiO_2_ on the cellular immune indices and body weight gains of calves [25]. It revealed that the examined feed additive did not significantly modulate the immune response of the calves and a beneficial effect of using this supplement in calves was observed in terms of a tendency towards higher average daily gains in the experimental animals when compared to the controls.

This research is concerned with the impact of the combination of two feed additives—one containing *L. edodes* mycelium enriched with Se in an organic form and the second containing protease and lipase (pancreatic-like enzymes of microbial origin), a fat-coated mixture of organic fumaric, malic, citric, and sorbic acids, sodium butyrate, and silicon nSiO_2_—on the serum Se concentration and selected immune parameters in calves. The average daily gains (ADG) of the calves were also evaluated. Before the start of administering the feed additives and during their delivery, the serum Se, immunoglobulin (Ig), and cytokine concentrations, WBC count with leukocyte differentiation, expression of LEU surface antigens, phagocytic activity, and oxidative burst of circulating MON and GRA were examined under experimental conditions.

## 2. Results

### 2.1. ADG of Calves

The ADG for the whole period of the experiment was 598 ± 141 g/d in the experimental (E) group and 574 ± 189 g/d in the control (C) group. All animals from the E group consumed the whole portions of both feed additives. The obtained differences were not statistically significant (*p* > 0.05).

### 2.2. Serum Se Concentration

The serum Se concentration in the E calves was significantly higher than in the C calves in weeks 1, 7, and 10 (Table 1).

### 2.3. Leukogram

**Figure 1:**
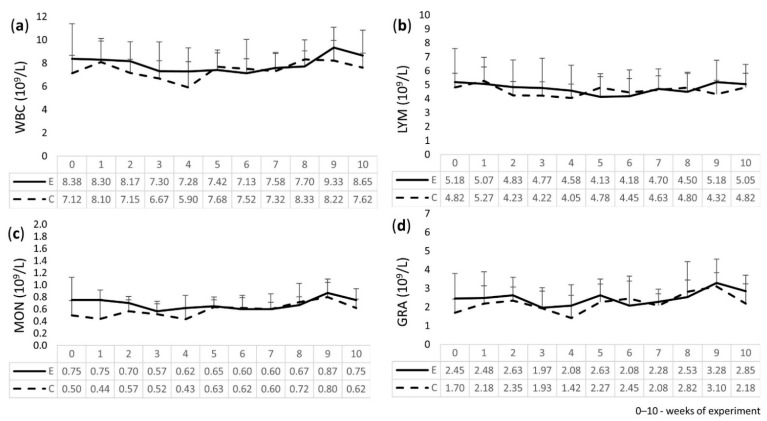
Results of examination of white blood cells (WBC) and its differentiation: (**a**) WBC; (**b**) lymphocytes (LYM); (**c**) monocytes (MON); (**d**) granulocytes (GRA), in the peripheral blood of experimental (E) and control (C) calves.

The results of the WBC, LYM, MON, and GRA analyses in the calves, as shown in Figure 1, revealed that all results were within the reference ranges [26] and no statistically significant differences were found between the E and C groups for all examined parameters, but slight differences were observed. Namely, there was a smaller decrease in the values of the WBC, LYM, MON, and GRA counts in the E group, and they were more normalized and higher in weeks 9 and 10 of the experiment when compared to the C group.

### 2.4. Immunophenotyping of LEU

**Figure 2:**
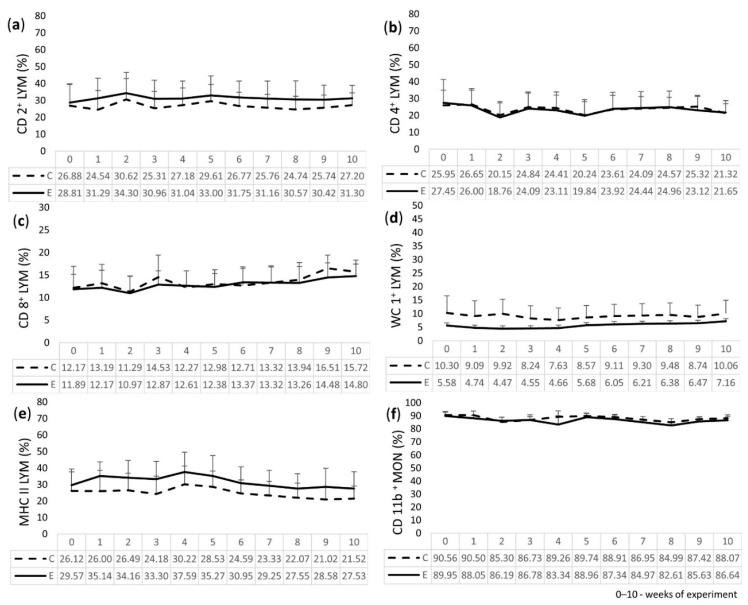
Percentage of the lymphocyte (LYM) subpopulations containing surface antigens in the peripheral blood of experimental (E) and control (C) calves: (**a**) CD2^+^; (**b**) CD4^+^; (**c**) CD8^+^; (**d**) WC1^+^; (**e**) MHC class II, and (**f**) percentage of the monocyte (MON) subpopulation containing CD11b^+^ surface antigens.

The results of cytometric analysis of the peripheral blood LEU of the calves are presented in Figure 2. For the E group, the percentage of CD2^+^ and MHC II (expressed on T LYM) was higher than for the C group throughout the experiment. WC1^+^ (gamma/delta T LYM) for the E group had lower values than those obtained for the C group, but it slightly increased throughout the time of the experiment. Regarding CD4^+^ (T helper LYM) and CD11b^+^ on peripheral blood MON, the values for the E group were similar to the C group, with the exception of lower values for CD4^+^ in week 9 and for CD11b^+^ in week 4. The percentage of CD8^+^ (T cytotoxic/suppressor LYM) for the E group was lower, with the exception of weeks 4 and 7, when the values were close to the C group, and week 6, when the value was higher. During the experiment, a tendency to decrease the percentage of CD4^+^ cells and to increase the percentage of CD8^+^ cells was observed for both groups. No statistically significant differences in the examined parameters were found between the E and C groups during the 10 weeks of the study.

### 2.5. Phagocytic Activity and Oxidative Burst of GRA and MON

#### 2.5.1. Phagocytic Activity of GRA and MON

The phagocytic activity of GRA and MON in the peripheral blood of calves and its mean fluorescence index (MFI) are presented in Figure S1. The percentage of phagocytic GRA in the E group was similar to the C group and normalized in the E group when compared to the C group. The results obtained for MON in the E group revealed that the values were comparable to the C group, but slight differences were observed. Namely, the percentage in the E group was lower in weeks 3, 4, and 9 and it was higher in weeks 7 and 8. The MFI values for phagocytic GRA in the E group were lower for weeks 2, 3, 7, and 8 and higher in weeks 1, 6, and 9 when compared to the C group, and in the remaining weeks of the study, the values were similar for both groups. The values for MON in the E group were lower than those obtained for the C group. For all values, no statistically significant differences (*p* > 0.05) were found between the E and C groups.

#### 2.5.2. Oxidative Burst of GRA and MON

The results of the oxidative burst activity of GRA and MON in the peripheral blood of the examined calves and its MFI are presented in Figure S2. In the E group, the percentage of GRA with oxidative burst activity was lower than the values obtained for the C group for most of the weeks, but it was comparable in weeks 5 and 8. No statistically significant differences were found between the E and C groups throughout the study. The values for MON in the E group were lower or comparable for most of the weeks of the study, while in the remaining time, i.e., weeks 3 and 7, it was higher. The MFI value for GRA with oxidative burst activity was lower in the E group for most of the weeks of the experiment, except for weeks 1 and 3, when the values were comparable with the C group. The MFI value for MON in the E group was higher in weeks 1, 2, 3, 9, and 10 when compared to the C group. In weeks 4 and 6, the values were lower, and in weeks 5, 7, and 8, they were comparable to the C group. For all values, no statistically significant differences were found between the E and C groups (*p* > 0.05).

### 2.6. Bovine Ig and Cytokine Concentrations in Calves’ Serum

**Figure 3:**
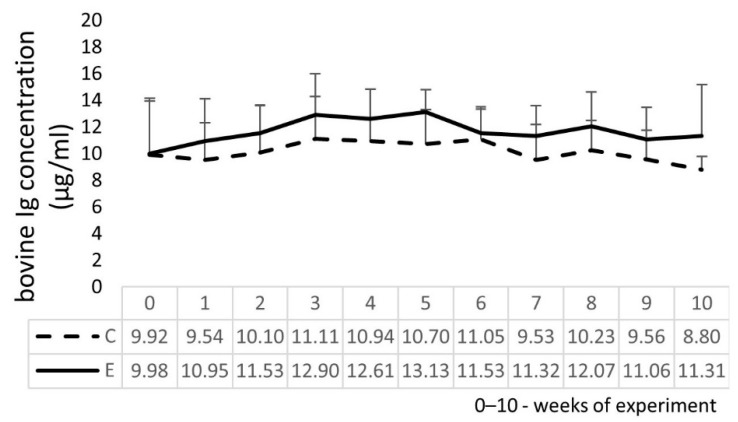
Bovine immunoglobulin (Ig) concentration in the serum of experimental (E) and control (C) calves.

The E group had a higher concentration of serum bovine Ig when compared to the C group (Figure 3), beginning from week 1 of the experiment until the end of the study. During the study, no changes in interferon γ (IFN γ), interleukin 1β (IL 1β), and tumor necrosis factor α (TNF α) concentrations were found, and they remained at zero levels in both the E and C groups of calves. The serum interleukin 2 (IL 2) concentration in three of six animals from the E group slightly increased from zero to the range of 109.4 pg/mL to 260.2 pg/mL from week 8 to 10, while the C group remained at zero value. Similarly, the interleukin 4 (IL 4) concentration in three of six animals from the E group had concentrations in the range of 32.9 pg/mL to 43.4 pg/mL in weeks 5 and 8, while IL 4 was not detected in the serum samples in the remaining weeks of the experiment. The interleukin 10 (IL 10) concentration in three of six E calves had concentrations in the range of 25.2 pg/mL to 64.0 pg/mL from week 7 to 10, while, in the remaining weeks of the experiment, IL 10 was not detected. The differences in the values between the E and C groups were not statistically significant (*p* > 0.05).

## 3. Discussion

The concept of supplementation of ruminant diets with a mixture of exogenous enzymes [27], acidifiers [28,29], sodium butyrate [30], and nSiO_2_ [31] is not new. It has been proven to obtain lower pH values in the digestive tract, ensuring the optimal pH for digestive enzymes, which improves nutrient digestibility. The above explains the tendency for higher AGD in calves fed with the feed additives examined in our study. Use of supplementation with the composition of ingredients listed above, examined in our previous study, was shown to have no adverse effect on the humoral immune response of calves [25].

Another aspect of cattle feeding is the Se deficiency connected with geographical conditions. In European countries, including Poland, Se deficiencies in the soil and, as a result, in animals fed with pasture grown on this soil constitute a significant problem [32,33]. For example, there are diagnosed Se deficiencies in calves from the Czech Republic [18]. Similarly, the calves enrolled in our study, both from the control and experimental groups, had serum Se concentrations below the reference value before the beginning of the experiment. The concentration increased to an adequate level (80–300 μg/L according to Puls 1988 [34]) after the first week of treatment with the examined feed additive and remained elevated until the end of the experiment, with lower values in weeks 1 and 10, when compared to week 7, but the concentration was within the reference range. Adequate Se concentration is advantageous for calves’ health and prevents them from acquiring deficiency-related diseases, including white muscle disease. It is important not to over-supplement this mineral because it can lead to Se poisoning in calves. These results are in accordance with a previous study [24], where the effect of a single ingredient—*L. edodes* mycelium enriched with selenitetriglycerides—offered to calves was evaluated. It revealed that Se administered to the calves at a higher dose than in this study—5 µg Se(IV)/kg b.w.—resulted in an increase in the Se concentration in the calves’ serum to the reference values. Our study proved that *L. edodes* mycelium enriched with Se was useful in significantly increasing the level of Se in the serum of calves, even though the dose was half that of the previous study. Research by Żarczyńska et al. [35] revealed that the concentration of Se in calves’ serum increased within one day of Se supplementation. In this experiment, Holstein-Friesian calves from two research groups (six animals in each group) were administered selenitetriglycerides once in the amount of 0.5 or 1 mg Se/kg b.w. on the second day of their lives, while the control calves were not supplemented.

The main objective of this study, beyond replenishing Se deficiencies, was to monitor parameters of the immune response in calves supplemented with a novel feed additive composed of different ingredients that are crucial for the proper functioning of its organism. To the best of the authors’ knowledge, there is no other comprehensive study concerning immune response evaluation during such a long period of administration of this combination of two feed additives. The Ig concentration is viewed as an indicator to assess the humoral immune response [36]. Our study reveals generally that the immune system is not negatively affected by the examined additives and an increase in the serum Ig concentration and alterations in leukocyte subsets in the E calves when compared to the C group, although not statistically significant, may contribute to fighting eventual infections.

More specific observations about the results of the analysis of the WBC and its’ subsets revealed only small fluctuations in the values between the E and C groups, but generally speaking, the blood parameters were not affected. These results agree with the findings from the study by Żarczyńska et al. [35] described above, in which a single oral administration of Se in the form of selenitetriglycerides did not influence the hematological parameters in the calves. Although pigs have different digestive tracts and diets, some conclusions can be drawn from the research by Lee et al. [37] on weaned, 28-day-old pigs. It revealed that the addition of 0.02% dietary protease can reduce the inflammatory immune response because the WBC in the experimental pigs was lower when compared to the negative control, which consisted of pigs that received a smaller amount of protein in the diet. Additionally, any changes in the WBC count were seen in the experimental pigs when compared to the positive control, i.e., animals fed with a standard diet.

The above findings regarding the hematological analysis in our study are reflected in the cytometric phenotyping of the peripheral blood leukocytes of the calves. The percentages of T lymphocytes and their subpopulations—T helper, T cytotoxic/suppressor, and γδ T LYM—as well as MHC II LYM, and the percentage of MON with the presence of β-integrin, were not significantly altered in the experimental calves that were fed with the feed additives. However, the cytometric analysis of MHC II surface antigens revealed a slightly higher percentage of MHC II on LYM of the E calves when compared to the C group. This is beneficial for the calves’ immunity because this kind of LYM takes part in the interface between the innate and adaptive immune systems, which is antigen presentation, and it results in specific T cell activation and a more efficient fight against pathogens. On the other hand, the cytometric analysis of the γδ T cells revealed that these cells have the ability to mediate immune functions by the secretion of specific cytokines, and regulate pathogen clearance in the host [38,39]. Our study revealed that the percentage of WC1^+^ cells, which are expressed on γδ T cells, was not elevated in the experimental calves. Moreover, their percentage was slightly lower in the experimental calves when compared to the control, so this population of leukocytes was not induced in the experimental conditions, i.e., while the examined feed additives were being fed to the calves.

The phagocytic activity and oxidative burst of LEU play a crucial role in the non-specific immune response, especially in host resistance against pathogens. In the conditions of this study, during supplementation with the additives, the proportions of GRA and MON that initiate phagocytic activity and oxidative burst were not compromised, nor was the intensity of these processes significantly altered during the 10 weeks of supplementation. This finding is in accordance with Chanput et al. [40], who examined β-glucan, a substance that is one of the main functional compounds of *L. edodes*. In their experiment, the phagocytic activity of macrophages from the human cell line was not changed after its stimulation by β-glucan derived from different products, i.e., oats, barley, and shiitake.

The examination of the serum cytokine concentrations in this study revealed unchanged pro-inflammatory cytokine concentrations. Moreover, increased anti-inflammatory cytokine concentrations in the serum of some of the E calves were observed in the latter period of the experiment. It can be assumed that a reduced inflammatory reaction can be observed during supplementation because there is a reduced amount of undigested feed in the gut, which prevents inflammatory reaction. This study is in accordance with the research on pigs by Lee et al. [37], described above, and we can conclude that the addition of the enzyme protease to the diet did not result in a higher TNF α concentration, one of the most important cytokines that stimulates the inflammatory response, in pigs’ serum [37]. Moreover, in our study, some protective effect was evidenced by the stimulation of the secretion of anti-inflammatory cytokines such as IL 2, IL 4, and IL 10 in some of the supplemented calves in the latter period of the experiment, although the differences between the groups were not statistically significant. There is no other study concerning cytokine expression after *L. edodes* enriched with Se supplementation, but we can conclude that the expression of anti-inflammatory cytokines is possible when using substances consisting of β-glucan, such as *L. edodes* mycelium. The above-mentioned research of Chanput et al. [40] shows that IL 10 gene expression was higher in macrophages after β-glucan stimulation, after 24 h of incubation, which is a late upregulation profile, and it means that this substance can exert anti-inflammatory properties under some conditions.

To summarize, supplementation of experimental calves with the combination of two feed additives—one containing the *L. edodes* mycelium enriched with Se in organic form, and the second containing a mixture of exogenous enzymes, acidifiers, sodium butyrate, and nSiO_2_—resulted in a significantly increased serum Se concentration in the calves, which was maintained at an adequate level throughout the study. The detailed analysis of the calves’ immune systems revealed that it was not suppressed throughout the period of delivery of the feed additives. Additionally, some positive effect was seen in terms of a tendency to increase some immune parameters, i.e., the serum Ig concentration, the percentage of the MHC II LYM subpopulation, and the serum anti-inflammatory cytokine concentrations. These findings, although not statistically significant, together with a tendency for higher ADG in the calves fed with both of the examined additives, confirmed a positive influence on the calves’ organisms. It is an essential prerequisite for future studies on the supplementation of calves with the examined feed additives on a larger scale, under farm conditions, to confirm these conclusions.

## 4. Materials and Methods

### 4.1. The Calves and Treatments

The experiment was conducted on twelve clinically healthy Holstein-Friesian female calves at an average age of 16.3 weeks (±5.9). It was performed in the vivarium of the National Veterinary Research Institute in Pulawy, Poland. The animals were randomly assigned to the E (*n* = 6) and C (*n* = 6) groups and housed in six pens (two animals per pen) with free access to water. The E and C animals were fed standard diets including milk replacer and calf starter feed in rising amounts (from 312.5 g to 375 g of milk replacer and from 500 g to 2000 g of calf starter feed) and received hay ad libitum. The detailed nutrient composition is shown in Table 2. Within the experiment, the calves from the E group were also supplemented with a novel composition of feed additives added to the morning portion of milk replacer once per day for the 10 weeks of the study. It contained *L. edodes* mycelium enriched with Se(IV) in the form of selenitetriglycerides at a dosage of 43 mg of selenium-enriched mushroom lyophilizate/kg b.w. of calves per day, i.e., 2.5 µg Se(IV)/kg b.w. of calves; 18 mg/per calf of protease and 45 mg/per calf of lipase; 250 mg/per calf of fumaric, 250 mg/per calf of malic, 120 mg/per calf of citric, and 220 mg/per calf of sorbic acids; 15 mg/per calf of sodium butyrate and 2860 mg/per calf of nSiO_2_, with a particle size of 5–10 nm and absorption area of 380 m^2^/g.

Throughout the experiment, the amount of feed intake and rectal temperature were recorded every day. The b.w. gains were recorded before the beginning of the experiment and for the 10 weeks of the study at weekly intervals. Blood samples were collected from the *vena jugularis externa* once per week during the experiment using single-needle insertion from each calf. For LEU counts, LYM subpopulation percentage analyses, and phenotyping of LYM, whole blood samples were collected in a 1 mL vacutainer with K2-EDTA as the anticoagulant (Medlab, Raszyn, Poland). For the phagocytic activity and oxidative burst analysis, whole blood samples were collected in a 2.5 mL vacutainer with heparin as the anticoagulant (Medlab, Raszyn, Poland). These samples were examined within 1 h. For cytokine expression analysis, whole blood samples were collected in plastic tubes and then centrifuged to obtain the sera for further analysis. The serum samples were stored under freeze conditions (−20 °C) before the analysis.

### 4.2. ADG of the Calves

ADG values were calculated by dividing the total b.w. gains, i.e., the difference between the calves’ maximum and minimum body weight, by the number of days of the experiment.

### 4.3. Serum Se Concentration

Se concentration in the serum samples of the E and C calves derived from weeks 0, 1, 7, and 10 of the experiment was analyzed by means of inductively coupled plasma mass spectrometry (ICP-MS) with the use of the Varian 820 MS, Bruker M 90, Plasma Quant ICP-MS (all from Analytik Jena, Jena, Germany), Varian Vista Pro (Agilent, Santa Clara, CA, USA), and iCAP Duo 7000 (ThermoFisher, Waltham, MA, USA) ICP optical emission spectrometers.

### 4.4. Leukogram

Total WBC counts with leukocyte differentiation, which included LYM, MON, and GRA, were examined in the peripheral blood using an Exigo automatic veterinary blood analyzer (Boule Medical AB, Spånga, Sweden).

### 4.5. Immunophenotyping of Leukocytes

A flow cytometer (BD FACSCalibur, Becton Dickinson, Franklin Lakes, NJ, USA) was used to determine the expression of surface antigens on LEU, i.e., CD2^+^, CD4^+^, CD8^+^, WC1^+^, MHC II, and CD11b^+^. The test was performed using single-cell labeling according to Muszyńska et al. [24]. The following mouse anti-bovine monoclonal antibodies (mAb) with fluorescein isothiocyanate (FITC) were used: CD2^+^, CD4^+^, CD8^+^, WC1^+^, MHC class II monomorphic, and CD11b^+^ (Bio-Rad, Hercules, USA). The tests were performed with 20,000 cells acquired for each experiment and analyzed with the CellQuest software (Becton Dickinson, Franklin Lakes, NJ, USA).

### 4.6. Phagocytic Activity and Oxidative Burst of GRA and MON

#### 4.6.1. Phagocytic Activity of GRA and MON

GRA and MON phagocytic activity was determined in whole blood samples using a commercial Phagotest kit (Glycotope Biotechnology, Heidelberg, Germany) according to Wójcicka-Lorenowicz et al. [41], with modifications. The percentage of GRA and MON that had phagocytized bacteria was evaluated. To estimate the individual cellular phagocytic activity, i.e., the amount of bacteria ingested per cell, the MFI of the phagocytizing cell population was assessed.

#### 4.6.2. Oxidative Burst of GRA and MON

The oxidative burst activity of GRA and MON after *E. coli* stimulation was examined in whole blood samples using a commercial Phagoburst kit (Glycotope Biotechnology, Heidelberg, Germany) according to Wójcicka-Lorenowicz et al. [41], with modifications. The oxygen metabolism of GRA and MON was determined using the percentage of cells phagocytizing *E. coli*. The MFI of this process, as the index of activity of cells with oxidative burst activity, was assessed. The samples were analyzed with the same equipment as above within 30 min.

### 4.7. Bovine Ig and Cytokine Concentrations in Calves’ Serum

The concentration of Ig was evaluated using a commercial ELISA kit (BioX Diagnostics, Rochefort, Belgium), and selected cytokines, i.e., IL 1β, TNF α, IFN γ, IL 2, IL 4, and IL 10, were evaluated using separate commercial ELISA kits (Cloud Clone Corporation, Katy, TX, USA). All tests were performed following manufacturers’ instructions. The optical densities for the analyzed parameters in the microwells of the plates were read at 450 nm using an automated plate reader (Elx800 Microplate Reader, BioTek Instruments, Inc., Winooski, VT, USA).

## 5. Statistical Analysis

All values are presented as arithmetic means ± standard deviation (SD). A repeated-measures ANOVA test was performed to check the main effects of experimental supplementation. A Tukey post hoc test was performed to check the differences between the groups (STATISTICA 10 software, StatSoft Inc., Tulsa, OK, USA). Regarding the comparison of ADG and serum Se concentration between the E and C groups, the significant differences between the mean values at each time point were analyzed using Student’s *t*-test. The effects were considered to be statistically significant at a level of *p* < 0.05.

## Figures and Tables

**Table 1:** Se concentration in serum of calves receiving the novel feed additive in diet (µg/L), mean ± SD.

Group of Calves	Week 0	Week 1	Week 7	Week 10
experimental	53.77 ± 5.54	90.15 ± 14.85 **	131.63 ± 18.02 **	92.03 ± 7.14 **
control	46.80 ± 11.90	45.68 ± 7.26	48.82 ± 5.12	52.00 ± 6.53

**—statistical significance at *p* < 0.001 with respect to the control calves.

**Table 2:** Ingredients and nutrient and chemical composition of the standard diet (on feed basis) of experimental and control calves.

Item	Milk Replacer	Calf Starter Feed
Soybeans (%)	31	N/A
Wheat flour (%)	30	N/A
Whey	28	N/A
Palm oil	9	N/A
Calcium carbonate	0.8	N/A
Dextrose	0.15	N/A
Beet molasses	0.05	N/A
Crude protein (%)	21.0	18.5
Crude oils and fats (%)	12.0	3.3
Ash (%)	6.0	9.0
Crude fiber (%)	1.1	6.5
Calcium (%)	0.7	1.3
Lysine (%)	1.4	-
Phosphorus (%)	0.5	0.8
Sodium (%)	0.3	0.23
Vitamin A (IU/kg)	10,000	25,000
Vitamin D3 (IU/kg)	2000	5000
Vitamin C (mg/kg)	100	-
Vitamin E (mg/kg)	80	25.0
Calcium D-pantothenate (mg/kg)	8.6	-
Niacinamide (mg/kg)	6.6	-
Vitamin B1 (mg/kg)	4.3	-
Vitamin B2 (mg/kg)	4.3	-
Vitamin B6 (mg/kg)	4.3	-
Vitamin K3 (mg/kg)	1.0	-
Folic acid (mg/kg)	0.33	-
Biotin (mg/kg)	0.07	-
Vitamin B12 (mg/kg)	0.05	-
Iron (mg/kg)	80	-
Manganese (mg/kg)	64	0.25 (%)
Zinc (mg/kg)	56	-
Copper (mg/kg)	8	-
Iodine (mg/kg)	0.96	-
Selenium (mg/kg)	0.2	-

N/A—not available.

## Data Availability

Data are contained within the article and Supplementary Materials.

## Supplementary Materials

The following supporting information can be downloaded at: https://www.mdpi.com/article/10.3390/molecules27165163/s1, Figure S1: Cytometric analysis of the phagocytic activity in the peripheral blood of experimental (E) and control (C) calves: (**a**) phagocytic activity of granulocytes (GRA) and monocytes (MON); (**b**) mean fluorescence intensity (MFI) as the number of bacteria absorbed by GRA and MON. Figure S2: Cytometric analysis of oxidative burst activity in the peripheral blood of experimental (E) and control (C) calves: (**a**) oxidative burst of granulocytes (GRA) and monocytes (MON); (**b**) mean fluorescence intensity (MFI) as the index of activity of cells with oxidative burst activity; *—statistical significance at *p* < 0.05 with respect to the control calves.

## Sample Availability

Samples of the compounds are not available from the authors.

## References

[1] Plattner B.L., Hostetter J.M. (2011). Comparative Gamma Delta T Cell Immunology: A Focus on Mycobacterial Disease in Cattle. Vet. Med. Int..

[2] Abbas A.K., Lichtman A., Pillai S. (2008). Cellular and Molecular Immunology.

[3] Quigley J.D., Drewry J.J., Murray L.M., Ivey S.J. (1997). Body Weight Gain, Feed Efficiency, and Fecal Scores of Dairy Calves in Response to Galactosyl-Lactose or Antibiotics in Milk Replacers. J. Dairy Sci..

[4] Sarker M., Ko S., Lee S., Kim G., Choi J., Yang C. (2010). Effect of Different Feed Additives on Growth Performance and Blood Profiles of Korean Hanwoo Calves. Asian-Australas. J. Anim. Sci..

[5] Ribeiro M.D., Pereira J.C., Queiroz A.C.D., Cecon P.R., Detmann E., Azevêdo J.A.G. (2009). Performance of dairy calves fed milk, milk replacer or post-weaning concentrate with acidifiers. Rev. Bras. Zootec..

[6] Płaczek A., Stępień P., Żarczyński P., Patorczyk–Pytlik B. (2019). Methods for enrichment of animal diets with selenium. J. Elem..

[7] Müller A., Bertram A., Freude B. (2014). Saisonale und überregionale Unterschiede im Selenversorgungsstatus von Rindern [Differences in the selenium supply of cattle across Europe]. Tierarztl. Prax. Ausg. G Grosstiere-Nutztiere.

[8] Kiremidjian-Schumacher L., Stotzky G. (1987). Selenium and immune responses. Environ. Res..

[9] Maplesden D.C., Loosli J.K. (1960). Nutritional Muscular Dystrophy in Calves. II. Addition of Selenium and Tocopherol to a Basal, Dystrophogenic Diet Containing Cod-Liver oil. J. Dairy Sci..

[10] Hefnawy A.E.G., Tórtora-Pérez J.L. (2010). The importance of selenium and the effects of its deficiency in animal health. Small Rumin. Res..

[11] Sordillo L.M. (2013). Selenium-dependent regulation of oxidative stress and immunity in periparturient dairy cattle. Vet. Med. Int..

[12] Fitak B., Grabowski M., Suchocki P. (1999). Preparat Przeciwnowotworowy i Sposób Jego Wytwarzania. Polish Patent.

[13] Pehrson B., Ortman K., Madjid N., Trafikowska U. (1999). The influence of dietary selenium as selenium yeast or sodium selenite on the concentration of selenium in the milk of suckler cows and on the selenium status of their calves. J. Anim. Sci..

[14] Ran L., Wu X., Shen X., Zhang K., Ren F., Huang K. (2010). Effects of selenium form on blood and milk selenium concentrations, milk component and milk fatty acid composition in dairy cows. J. Sci. Food Agric..

[15] Suchocki P., Misiewicz-Krzemińska I., Skupińska K., Niedźwiecka K., Lubelska K., Fijałek Z., Kasprzycka-Guttman T. (2010). Selenitetriglicerydes affect CYP1A1 and QR activity by involvement of reactive oxygen species and Nrf2 transcription factor. Pharmacol. Rep..

[16] Guo F.C., Williams B.A., Kwakkel R.P., Li H.S., Li X.P., Luo J.Y., Li W.K., Verstegen M.W.A. (2004). Effects of mushroom and herb polysaccharides, as alternatives for an antibiotic, on the cecal microbial ecosystem in broiler chickens. Poult. Sci..

[17] Turlo J., Gutkowska B., Herold F., Dawidowski M., Słowiński T., Zobel A. (2010). Relationship between selenium accumulation and mycelial cell composition in *Lentinula edodes* (Berk.) cultures. J. Toxicol. Environ. Health Part A.

[18] Ksiazek I., Sitarz K., Roslon M., Anuszewska E., Hoser G., Dudkiewicz-Wilczyńska J., Iwanowska M., Suchocki P. (2014). The influence of an organic selenium(IV) compound on progression of tumor induced using prostate cancer cells and gene expression connected to the oxidative stress response. World J. Pharm. Sci..

[19] Drori A., Shabat Y., Ya’acov A.B., Danay O., Levanon D., Zolotarov L., Ilan Y. (2016). Extracts from *Lentinula edodes* (Shiitake) edible mushrooms enriched with vitamin D exert an anti-inflammatory hepatoprotective effect. J. Med. Food.

[20] Muszyńska B., Bederska D., Zięba P. (2018). The importance of selenium in the human diet—in the aspect of feeding farm animals. Rocz. Nauk. Zootech..

[21] Murphy E.A., Davis J.M., Carmichael M.D. (2010). Immune modulating effects of β-glucan. Curr. Opin. Clin. Nutr. Metab. Care.

[22] Muszyńska B., Kała K., Włodarczyk A., Krakowska A., Ostachowicz B., Gdula-Argasinska J., Suchocki P. (2020). *Lentinula edodes* as a source of bioelements released into artificial digestive juices and potential anti-inflammatory material. Biol. Trace Elem. Res..

[23] Friedman M. (2016). Mushroom Polysaccharides: Chemistry and Antiobesity, Antidiabetes, Anticancer, and Antibiotic Properties in Cells, Rodents, and Humans. Foods.

[24] Muszyńska B., Szacawa E., Bederska-Łojewska D., Dudek K., Pomierny B., Włodarczyk A., Kała K., Lazur J., Suchocki P., Budziszewska B. (2020). Preliminary study on Se-enriched *Lentinula edodes* mycelium as a proposal of new feed additive in selenium deficiency. PLoS ONE.

[25] Szacawa E., Dudek K., Bednarek D., Pieszka M., Bederska-Łojewska D. (2021). A pilot study on the effect of a novel feed additive containing exogenous enzymes, acidifiers, sodium butyrate and silicon dioxide nanoparticles on the selected cellular immune indices and body weight gains of calves. J. Vet. Res..

[26] Jain N.C. (1993). Essentials of Veterinary Hematology.

[27] Guilloteau P., Plodari M., Romé V., Savary G., Le Normand L., Zabielski R. (2011). Pancreatic enzyme deficiency depends on dietary protein origin in milk-fed calves. J. Dairy Sci..

[28] Pearlin B.V., Muthuvel S., Govidasamy P., Villavan M., Alagawany M., Farag M.R., Dhama K., Gopi M. (2020). Role of acidifiers in livestock nutrition and health: A review. J. Anim. Physiol. Anim. Nutr..

[29] Janik A., Pieszka M. (2006). Effectiveness of probiotic, acidifier and mannan oligosaccharide use in piglet rearing. Ann. Anim. Sci. Suppl..

[30] Hill T.M., Aldrich J.M., Schlotterbeck R.L., Bateman H.G. (2007). Effects of changing the fat and fatty acid composition of milk replacers fed to neonatal calves. Prof. Anim. Sci..

[31] Szczurek P., Kamyczek M., Pierzynowski S., Goncharova K., Michałowski P., Weström B., Pryhodko O., Grabowski T., Pieszka M. (2016). Effects of dietary supplementation with pancreatic-like enzymes of microbial origin (PLEM) and silicon dioxide (SiO_2_) on the performance of piglets fed creep feed. J. Anim. Sci..

[32] Khanal D., Knight A.P. (2010). Selenium: Its role in livestock health and production. J. Agric. Environ..

[33] Patorczyk-Pytlik B., Kulczycki G. (2009). Content of selenium in arable soils near Wroclaw. J. Elem..

[34] Puls R. (1988). Mineral Levels in Animal Health: Diagnostic Data.

[35] Żarczyńska K., Sobiech P., Tobolski D., Mee J.F., Illek J. (2021). Effect of a single, oral administration of selenitetriglycerides, at two dose rates, on blood selenium status and haematological and biochemical parameters in Holstein-Friesian calves. Ir. Vet. J..

[36] Deng Z.Y.J., Zhang W., Wu G.Y., Yin Y., Ruan Z., Li T.J., Chu W.Y., Kong X.F., Zhang Y.M., Fan Y.W. (2007). Dietary supplementation with polysaccharides from Semen cassiae enhances immunoglobulin production and interleukin gene expression in early-weaned piglets. J. Sci. Food Agric..

[37] Lee J.J., Kang K., Park S., Cho J.H., Oh S., Park D.-J., Perez-Maldonado R., Cho J.-Y., Park I.-H., Kim H.B. (2020). Effects of dietary protease on immune responses of weaned pigs. J. Anim. Sci. Technol..

[38] García V.E., Sieling P.A., Gong J., Barnes P.F., Uyemura K., Tanaka Y., Bloom B.R., Morita C.T., Modlin R.L. (1997). Single-cell cytokine analysis of gamma delta T cell responses to nonpeptide mycobacterial antigens. J. Immunol..

[39] O’Brien R.L., Roark C.L., Jin N., Aydintug M.K., French J.D., Chain J.L., Wands J.M., Johnston M., Born W.K. (2007). The gamma-delta T-cell receptors: Functional correlations. Immunol. Rev..

[40] Chanput W., Reitsma M., Kleinjans L., Mes J.J., Savelkoul H.F., Wichers H.J. (2012). β-Glucans are involved in immune-modulation of THP-1 macrophages. Mol. Nutr. Food Res..

[41] Wojcicka-Lorenowicz K., Kostro K., Lisiecka U., Gąsiorek B. (2018). Phagocytic activity and oxygen metabolism of peripheral blood granulocytes from rabbits experimentally infected with *Trichophyton mentagrophytes*. J. Vet. Res..

