# Supplementary figures and images for: Effect of Supplementation with the Combination of Se-Enriched Lentinula edodes Mycelium, Exogenous Enzymes, Acidifiers, Sodium Butyrate and Silicon Dioxide Nanoparticle Feed Additives on Selected Parameters in Calves
Source: Molecules. 2022 Aug 13;27(16):5163. doi: 10.3390/molecules27165163 (PMC9413389; doi:10.3390/molecules27165163)

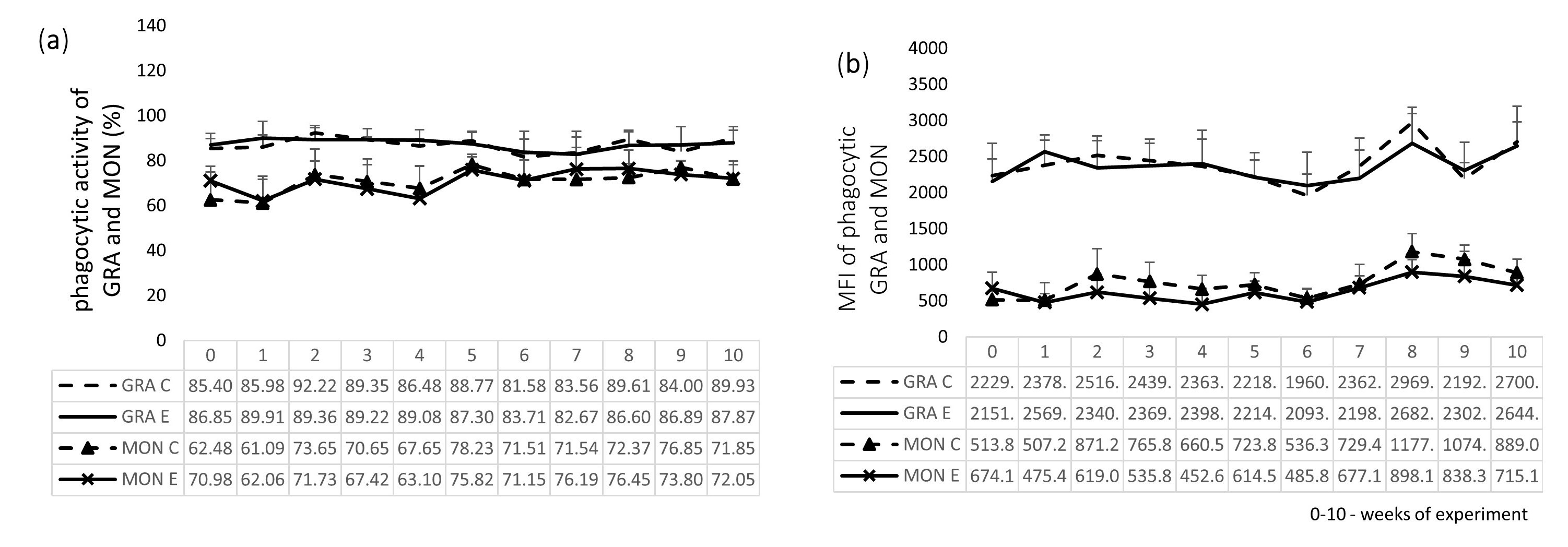

Supplement: Supplementary file 1 [file molecules-27-05163-s001.zip › Figure S1.JPG]

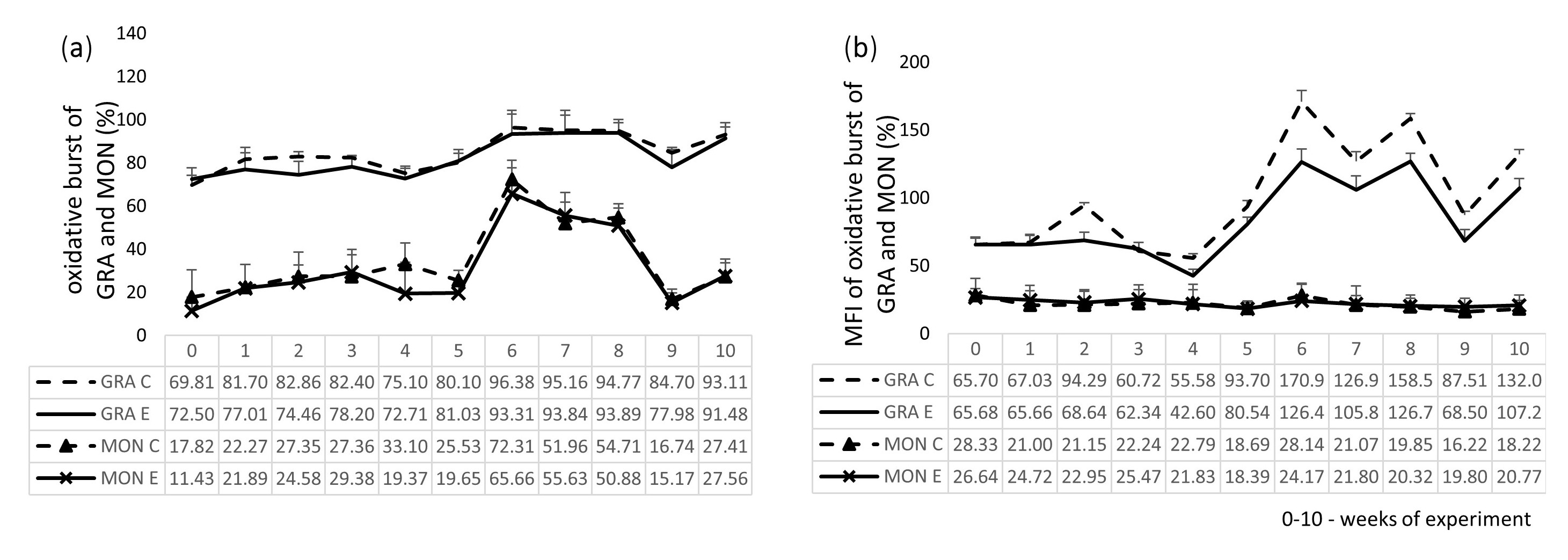

Supplement: Supplementary file 1 [file molecules-27-05163-s001.zip › Figure S2.JPG]
